# Correction: Circulating microRNAs as potential biomarkers of early vascular damage in vitamin D deficiency, obese, and diabetic patients

**DOI:** 10.1371/journal.pone.0315309

**Published:** 2024-12-05

**Authors:** Adel B. Elmoselhi, Mohamed Seif Allah, Amal Bouzid, Zeinab Ibrahim, Thenmozhi Venkatachalam, Ruqaiyyah Siddiqui, Naveed Ahmed Khan, Rifat A. Hamoudi

There are errors in the author affiliations. The correct affiliations are as follows:

Adel B. Elmoselhi^1,2^, Mohamed Seif Allah^1,3^, Amal Bouzid^2^, Zeinab Ibrahim^2^, Thenmozhi Venkatachalam^2^, Ruqaiyyah Siddiqui^4,5^, Naveed Ahmed Khan^1,2,5^, Rifat A. Hamoudi^1,2^

**1** College of Medicine, University of Sharjah, Sharjah, UAE, **2** Sharjah Institute of Medical Research, College of Medicine, University of Sharjah, Sharjah, UAE, **3** Cardiology Department, University Hospital Sharjah, Sharjah, UAE, **4** College of Arts and Sciences, American University of Sharjah, University City, Sharjah, UAE, **5** Department of Medical Biology, Faculty of Medicine, Istinye University, Istanbul, 34010, Turkey.

## References

[1] Elmoselhi AB, Seif Allah M, Bouzid A, Ibrahim Z, Venkatachalam T, Siddiqui R, et al. (2023) Circulating microRNAs as potential biomarkers of early vascular damage in vitamin D deficiency, obese, and diabetic patients. PLOS ONE 18(3): e0283608. 10.1371/journal.pone.0283608 36952563 PMC10035929

