# Supplementary material for: Circulating microRNAs as potential biomarkers of early vascular damage in vitamin D deficiency, obese, and diabetic patients
Source: PLoS One. 2023 Mar 23;18(3):e0283608. doi: 10.1371/journal.pone.0283608 (PMC10035929; doi:10.1371/journal.pone.0283608)
Supplement: Attachment — Submitted filename: Response to Reviewers.docx [file pone.0283608.s001.docx]

**Reviewer #1:**

Elmoselhi et al submit an original research article entitled "Circulating MicroRNAs as Potential Biomarkers of Early Vascular Damage in Vitamin D Deficiency, Obese, and Diabetic Patients". They looked at the potential of seric miRNAs as biomarkers of Vitamin D3 deficiency, obesity, and diabetes melitus in 23 middle-aged Emiratis patients. This cohort is very limited. They used a whole transcriptome analysis with qPCR validation for miRNA in plasma samples. They conclude that miR-182-5p; miR-199a-5p; miR-193a-5p; and miR-155-5p were differentially over-expressed in high-risk patients for CVD vs healthy controls.

Thank you so much for your valuable comments and feedback. Please find below our response to your comments which will improve our manuscript. We agreed of the small cohort of the study; however, we have addressed the reasons in the methods (Lines 161-164) and put it as a limitation in the discussion (Lines 398- 401).

**Comments:**

-the C1 group (control) is described as having a normal BMI yet they are at a an average of 26 so above the normal level. Can the authors comment?

Thank you very much for pointing this out. Yes, there are 4 individuals in the control group who have BMI slightly about 25; however, our cut off for obese and non-obese is BMI ≥ 30 and BMI < 30 respectively. Therefore, we now change the control group criteria as BMI below 30, not normal BMI or non-obese individuals. It is noteworthy to mention that at this age group, it is more difficult to find people with normal BMI among other criteria such as normal vitamin D level, no previous CVDs, etc. We have corrected the text accordingly in lines: 39, 137, 159, 212, 254, 302, 308, 314, 343.

-What controls were performed to check quality of RNAs after extraction?

Thank you for your comment. Yes, the spectrophotometry was performed to measure the absorbance of light by the RNA samples at different wavelengths. The ratio of absorbance at 260 nm and 280 nm (A260/A280) is used to assess the purity of RNA. A ratio of around 2.0 indicates pure RNA without protein or organic contamination. This sentence has been added in the methods section (Lines 197-199).

-Can the authors justify the use of miR-19b-3p as housekeeping miRNA? Can the authors show that this miRNA is the same in all groups?

MiR-19b-3p has been reported to be stably expressed in different tissues and cell types, making it a suitable candidate for normalization of miRNA expression data. Several studies have shown that miR-19b-3p expression is relatively constant across different samples and experimental conditions (1, 2). A general statement with references has been added in the methods section (Lines 294-295).

1. Song X, Li W, Shen P, Bai B, Cao LL. miR-19 Is a Potential Clinical Biomarker for Gastrointestinal Malignancy: A Systematic Review and Meta-analysis. Biomed Res Int. 2020 Sep 10;2020:2810150. doi: 10.1155/2020/2810150. PMID: 32964023; PMCID: PMC7501555.
2. Duca RB, Massillo C, Dalton GN, Farré PL, Graña KD, Gardner K, De Siervi A. MiR-19b-3p and miR-101-3p as potential biomarkers for prostate cancer diagnosis and prognosis. Am J Cancer Res. 2021 Jun 15;11(6):2802-2820. PMID: 34249429; PMCID: PMC8263646.

-A recapitulative final figure would be useful

The final figure has been adjusted to summarize the effect of vitamin D deficiency, obesity and DM on the early damage of blood vessels via expression of specific miRNAs and signaling pathways. Thank you.
